# 

**Published:** 1956-01

**Authors:** 


### THE MEDICAL JOURNAL OF THE SOUTH-WEST

*Incorporating*
*The Bristol Medico-Chirurgical Journal*

PUBLISHED UNDER THE AUSPICES OF THE
BRISTOL MEDICO-CHIRURGICAL SOCIETY

1956 *Vol.* 71

J. W. ARROWSMITH LTD., WINTERSTOKE ROAD, BRISTOL

### 1956 Contents of Vol. 71

| | page |
|---|---|
| Editorial: Dr. Robert Hughes Parry | 1 |
| Congenital Bladder-Neck Obstruction. By ASHTON MILLER, M.A., M.D., F.R.C.S. AND J. P. MITCHELL, M.S., F.R.C.S. | 3 |
| Cancer of the Uterus. By G. GORDON LENNON, CH.M., F.R.C.O.G., M.M.S.A. | 15 |
| Telling the Public about Medicine. By I. HARVEY FLACK, M.D. | 23 |
| Caleb Parry of Bath. By JOHN APLEY | 30 |
| Primary Myelosclerosis with Leuco-Erythroblastic Anaemia (A Clinico-Pathological Report) | 33 |
| Vital Statistics of the South-West in 1954. By A. H. GALE, D.M. | 39 |
| A Morbid View of the Over-65's. By G. STEWART SMITH, M.A., M.D. | 44 |
| The Position of Nursing in Hospitals in Bristol. By STEPHEN C. MERIVALE | 55 |
| Paediatric Comment. By A.V.N. | 58 |
| Perspective in Paediatrics. By Professor W. S. CRAIG | 59 |
| The New Outlook in Oto-Laryngology. By R. SCOTT STEVENSON, M.D., F.R.C.S.E.D. | 67 |
| Maternal Death Due to Rheumatic Heart Disease (A Clinico-Pathological Report) | 79 |
| Medical Worthies of the West County. By PERCY PHILLIPS, M.D., CH.B., M.SC. | 87 |
| The Growth of Human Tumours in Laboratory Animals. By G. J. HADFIELD, M.S., F.R.C.S. | 94 |
| Domestic Deaths from Electrocution. By F. E. CAMPS | 96 |
| Space Medicine. By S. G. FLAVELL MATTS, M.B., M.R.C.S. | 103 |
| Hypercalcaemia with Hypocalcuria: An Unusual Case of Primary Hyperparathyroidism. By H. A. W. FORBES AND M. J. WODZINSKI | 107 |
| Generalized Osteitis Fibrosa with Parathyroid Adenoma (A Clinico-Pathological Report) | 112 |
| Editorial: Visiting Professors | 119 |
| Visit to Iraq and Turkey. By Professor G. GORDON LENNON | 120 |
| Student Apprenticeship to General Practitioners. By W. M. CAPPER, F.R.C.S. | 124 |
| Modern Trends in Public Health. By R. C. WOFINDEN, M.D., D.P.H. | 126 |
| Domenique Jean Larrey, 1766–1842. By A. E. S. ROBERTS | 134 |
| "Eunuchs do not take the Gout." By G. D. KERSLEY, T.D., M.D., F.R.C.P. | 136 |
| Islet Cell Tumour of the Pancreas (A Clinico-Pathological Report) | 138 |
| Obituary: Arthur Harold Gale, M.D., D.P.H. | 142 |
| CORRESPONDENCE | 82 |
| ANNOTATION | 83 |
| NEWS FROM SOCIETIES | 83, 143 |
| APPOINTMENTS | 37, 85, 116, 148 |
| NOTES AND NEWS | 84, 115, 142 |

Medical Journal of the South-West.

### 1956 Index to Vol. 71

Apley, John.—Caleb Parry of Bath, 30

Caleb Parry of Bath.—John Apley, 30

Camps, F. E.—Domestic Deaths from Electrocution, 96

Cancer of the Uterus.—G. Gordon Lennon, 15

Capper, W. M.—Student Apprenticeship to General Practitioners, 124

Clinico-Pathological Reports:

—Primary Myelosclerosis with Leuco-Erythroblastic Anaemia, 33

—Maternal Death due to Rheumatic Heart Disease, 79

—Generalized Osteitis Fibrosa with Parathyroid Adenoma, 112

—Islet Cell Tumour of the Pancreas, 138

Congenital Bladder-Neck Obstruction.—Ashton Miller and J. P. Mitchell, 3

Craig, Prof. W. S.—Perspective in Paediatrics, 59

Domestic Deaths from Electrocution.—F. E. Camps, 96

Editorials:

—Dr. Robert Hughes Parry, 1

—Visiting Professors, 119

"Eunuchs do not take the Gout."—G. D. Kersley, 136

Flack, I. Harvey.—Telling the Public about Medicine, 23

Forbes, H. A. W., and Wodzinski, M. J.—Hypercalcaemia with Hyporcalcuria: an Unusual Case of Primary Hyperparathyroidism, 107

Gale, A. H.—Vital Statistics of the South-West in 1954, 39

Gale, Arthur Harold, M.D., D.P.H. (Obituary), 142

Generalized Osteitis Fibrosa with Parathyroid Adenoma—a Clinico-Pathological Report, 112

Growth of Human Tumours in Laboratory Animals, The.—G. J. Hadfield, 94

Hadfield, G. J.—The Growth of Human Tumours in Laboratory Animals, 94

Hypercalcaemia with Hypocalcuria: an Unusual Case of Primary Hyperparathyroidism.—H. A. W. Forbes and M. J. Wodzinski, 107

Islet Cell Tumour of the Pancreas—a Clinico-Pathological Report, 138

Kersley, G. D.—"Eunuchs do not take the Gout," 136

Lavrey, Domenique Jean, 1766–1842.—A. E. S. Roberts, 134

Lennon, G. Gordon.—Cancer of the Uterus, 15

Lennon, G. Gordon.—Visit to Iraq and Turkey, 120

Maternal Death due to Rheumatic Heart Disease—a Clinico-Pathological Report, 79

Matts, S. G. Flavell.—Space Medicine, 103

Medical Worthies of the West Country.—Percy Phillips, 87

Merivale, Stephen C.—The Position of Nursing in Hospitals in Bristol, 55

Miller, Ashton, and Mitchell, J. P.—Congenital Bladder-Neck Obstruction, 3

Mitchell, J. P., and Miller, Ashton.—Congenital Bladder-Neck Obstruction, 3

Modern Trends in Public Health.—R. C. Wofinden, 126

Morbid View of the Over-65's.—A. G. Stewart Smith, 44

N., A. V.—Paediatric Comment, 58

New Outlook in Oto-Laryngology, The—R. Scott Stevenson, 67

Obituary: Arthur Harold Gale, M.D., D.P.H., 142

Paediatric Comment.—A.V.N., 58

Parry, Dr. Robert Hughes (Editorial), 1

Perspective in Paediatrics.—Prof. W. S. Craig, 59

Phillips, Percy.—Medical Worthies of the West Country, 87

Position of Nursing in Hospitals in Bristol, The.—Stephen C. Merivale, 55

Primary Myelosclerosis with Leuco-Erythroblastic Anaemia—a Clinico-Pathological Report, 33

Professors, Visiting (Editorial), 119

Roberts, A. E. S.—Domenique Jean Larrey, 1766–1842, 134

Smith, G. Stewart.—A Morbid View of the Over-65's, 44

Space Medicine.—S. G. Flavell Matts, 103

Stevenson, R. Scott.—The New Outlook in Oto-Laryngology, 67

Student Apprenticeship to General Practitioners.—W. M. Capper, 124

Telling the Public about Medicine.—I. Harvey Flack, 23

Visit to Iraq and Turkey.—G. Gordon Lennon, 120

Vital Statistics of the South-West in 1954.—A. H. Gale, 39

Wodzinski, M. J., and Forbes, H. A. W.—Hypercalcaemia with Hypocalcuria: an Unusual Case of Primary Hyperparathyroidism, 107

Wofinden, R. C.—Modern Trends in Public Health, 126

THE

MEDICAL JOURNAL OF THE SOUTH-WEST

*(Incorporating the Bristol Medico-Chirurgical Journal)*

Established 1883

VOL. 71 (i) | January, 1956 | NO. 259

### Contents

| | | Page |
|---|---|---|
| DR. ROBERT HUGHES PARRY | *Editorial* | 1 |
| CONGENITAL BLADDER-NECK OBSTRUCTION: | | |
| PART I | *Mr. J. P. Mitchell* | 3 |
| PART II | *Mr. Ashton Miller* | 10 |
| CANCER OF THE UTERUS | *Prof. G. Gordon Lennon* | 15 |
| TELLING THE PUBLIC ABOUT MEDICINE | *Dr. I. Harvey Flack* | 23 |
| CALEB PARRY OF BATH | *Dr. John Apley* | 30 |
| PRIMARY MYELOSCLEROSIS WITH LEUCO-ERYTHROBLASTIC ANAEMIA | *Clinical Pathological Conference* | 33 |
| APPOINTMENTS | | 37 |

*PUBLISHED QUARTERLY*

*ANNUAL SUBSCRIPTION* **16/-** *POST FREE*

PUBLISHED UNDER THE AUSPICES OF THE
BRISTOL MEDICO-CHIRURGICAL SOCIETY

### *Editorial Committee*

Dr. J. E. CATES, Department of Medicine, Bristol Royal Infirmary. *Editor.*
Dr. A. H. GALE, The University, Bristol 8. *Assistant Editor.*
Dr. J. MACRAE, Ham Green Hospital. *Treasurer.*
Dr. N. J. BROWN, Southmead Hospital, Bristol.
Mr. J. P. PARTRIDGE, Southmead Hospital, Bristol.

### *Local Correspondents*

*Bath* . . Mr. A. D. BATEMAN, 3 The Circus, Bath.
*Exeter* . . Dr. L. N. JACKSON, Mount Jocelyn, Crediton, Devon.
*Gloucester* . Dr. R. F. JARRETT, 9 College Green, Gloucester.
*Plymouth* . Dr. C. R. CROFT, Lexden, Hartley Av., Plymouth.
*Taunton* . Dr. M. MCALLEY, 1 The Crescent, Taunton.
*Torquay* . Dr. A. ROBINSON THOMAS, 20 Devon Square, Newton Abbot, Devon.
*Truro* . . Dr. F. D. M. HOCKING, St. Andrews, Perranporth, Cornwall.
*Yeovil* . . Mr. J. A. VERE NICOLL, Knapp House, Yeovil, Somerset.

### NOTICE TO CONTRIBUTORS

Original articles are invited, provided they have not been published elsewhere. Notes of forthcoming events, meetings held, degrees conferred, staff appointments, and other local news are welcomed. The editorial committee reserves the right to make literary corrections.

Illustrations should always be supplied ready for reproduction. All re-touchings or other operations required will be charged to the author. Line drawings should be drawn in Indian ink. We regret that we must ask authors to pay for making blocks, if this is necessary.

J. W. ARROWSMITH LTD., WINTERSTOKE ROAD,
BRISTOL.

### *Appointments*

### *UNITED BRISTOL HOSPITALS*

| *Name* | *Appointment* | *Formerly* |
|---|---|---|
| FREEMAN, J., M.B., B.S. (LOND.), F.R.C.S. (ENG.) | Consultant Surgeon (E.N.T.). | Senior Registrar (E.N.T.) United Bristol Hospitals. |

### SENIOR REGISTRAR AND REGISTRAR APPOINTMENTS

| | | |
|---|---|---|
| MILLS, C. P., M.B., B.S. (LOND.), F.R.C.S. (ENG.). | Senior Registrar (E.N.T.). | Registrar (E.N.T.). |
| WILLIAMS, R. A., M.B., B.CHIR., D.L.O. | Registrar (E.N.T.). | S.H.O. (E.N.T.), Westminster Hospital. |
| WESLEY-JAMES, O., M.B., B.S. (LOND.). | Registrar (Casualty and General Surgery). | Surgical Registrar, University College Hospital, Ibadan. |
| VERO, D., L.D.S., R.C.S. (ENG.). | Registrar Dental Surgery. | In charge of " English Electric " Dental Clinic, Stafford. |

### *SOUTH-WESTERN REGIONAL HOSPITAL BOARD*

### CONSULTANTS, SPECIALISTS AND REGISTRARS APPOINTED DURING THE MONTHS OF OCTOBER AND NOVEMBER, 1955

### *BRISTOL*

| *Name* | *Appointment* | *Formerly* |
|---|---|---|
| TOVEY, L. A. D., M.B., CH.B. (BRISTOL). | Re-appointed for further year as Registrar to the South West Regional Blood Transfusion Service, Southmead. | In same post. |
| VARMA, R. M., M.B., B.S. (MADRAS), PRIMARY F.R.C.S. (EDIN.) AND (ENG.). | Registrar to the Department of Neurological Surgery, Frenchay Hospital. | Surgical Registrar, Royal Cornwall Infirmary, Truro. |
| SEAGER, C. P., B.SC. (WALES), M.B., B.CH. (WALES), D.P.M. (CONJOINT). | Senior Registrar in Psychiatry, Bristol Mental Hospitals. | Psychiatric Registrar, Belmont Hospital, Sutton, Surrey. |
| THOMSON, J. L. G., M.B., B.S. (LOND.), D.M.R.D., M.D. (LOND.), M.R.C.P. (LOND.). | Consultant Radiologist to the Bristol Clinical Area (Frenchay Hospital). | Senior Registrar in Radiodiagnosis, United Oxford Hospitals. |

### *NORTH GLOUCESTERSHIRE*

| *Name* | *Appointment* | *Formerly* |
|---|---|---|
| ELRICK, W. L., M.B., B.S. (MELBOURNE), PRIMARY F.R.A.C.S. | Surgical Registrar, Cheltenham General, Eye and Children's Hospital. | Undertaking Final Fellowship course at Guy's Hospital. |
| MERRYWEATHER, R., M.B., B.S. (LOND.), F.R.C.S. (ENG.). | Consultant Orthopaedic Surgeon, North Gloucestershire Clinical Area. | Senior Orthopaedic Registrar, Exeter Clinical Area. |
| MANDELBROTE, B., M.B., CH.B. (CAPE TOWN), M.R.C.P. (LOND.), D.P.M. (CONJOINT). | Medical Superintendent, Horton Road and Coney Hill Hospitals, Gloucester. | Assistant Psychiatrist, Warlingham Park Hospital. |

### *BATH*

| *Name* | *Appointment* | *Formerly* |
| --- | --- | --- |
| HABIB, G. G., B.SC. (B'HAM), M.B., CH.B. (B'HAM.). | Medical Registrar to the Bath Group of Hospitals. | Senior House Officer in Clinical Pathology, St. George's Hospital, London. |

### *SOUTH SOMERSET*

| *Name* | *Appointment* | *Formerly* |
| --- | --- | --- |
| GRAHAM, A. J., M.B., CH.B. (ABERDEEN), D.P.M. (CONJOINT). | Senior Registrar in Psychiatry, Tone Vale Hospital, near Taunton. | Psychiatric Registrar, Graylingwell Hospital, Chichester. |

### *DEVON AND CORNWALL*

| *Name* | *Appointment* | *Formerly* |
| --- | --- | --- |
| YOUINGS, M. D., M.B., B.S. (LOND.). | S.H.M.O. in Infectious Diseases, Scott Isolation Hospital, Plymouth. | J.H.M.O. at same hospital. |
| AKEHURST, A. C., M.B., B.S. (LOND.), F.R.C.S. (ENG.). | Senior Surgical Registrar, Exeter Clinical Area. | Surgical Registrar, United Bristol Hospitals. |
| SINGHA, H. S., M.A., M.B., B.CHIR. (CANTAB.), F.R.C.S. (EDIN.), AND (ENG.). | Surgical Registrar, South Devon and East Cornwall Hospital, Plymouth. | Junior Registrar, Surgical Outpatients, The London Hospital. |

